# Psychosocial-spiritual interventions among Muslims undergoing treatment for cancer: an integrative review

**DOI:** 10.1186/s12904-021-00746-x

**Published:** 2021-03-29

**Authors:** Abdallah Abu Khait, Mark Lazenby

**Affiliations:** 1 Department of Community and Mental Health Nursing, Faculty of Nursing, The Hashemite University, P.O. Box 330127, Zarqa, 13133 Jordan; 2 School of Nursing, University of Connecticut, Storrs, CT USA

**Keywords:** Psychotherapy, Muslim patients, Cancer, Psychosocial-spiritual outcomes

## Abstract

**Background:**

Cancer is a global disease that affects all populations, including Muslims. Psychological and spiritual care of Muslim patients with cancer includes psychosocial and spiritual techniques that help to improve their mental health and spiritual well-being. Although these techniques are available to cancer patients worldwide, they are poorly studied among Muslim patients. This review aims to integrate the literature on the psychosocial-spiritual outcomes and perspectives of Muslim patients with cancer who have undergone psychotherapy.

**Method:**

We used the Whittemore and Knafl five-step methodology. We conducted a comprehensive search of PubMed, CINAHL, and PsycINFO using relevant keywords. Studies that focused on adult patients with cancer and on published evidence of using psychosocial and spiritual interventions among Muslim patients were included. Each study was reviewed, evaluated, and integrated.

**Results:**

A systematic search produced 18 studies that were thematically analyzed. Results showed different psychosocial and spiritual approaches currently used to care for Muslim patients with cancer that target mainly patients’ mental health, quality of life, and spiritual well-being. Four major themes emerged: (1) *Treating Psychological Distress Without Psychopharmacologic Agents*, (2) *Improving Knowledge of Cancer for Improving QOL*, (3) *Depending on Faith for Spiritual Well-being*, and (4) *Relying on Religious and Spiritual Sources: Letting Go, Letting God*.

**Conclusions:**

The rigor of psychosocial and spiritual studies that target psychosocial-spiritual outcomes of Muslim cancer patients needs to be improved to reach conclusive evidence about their efficacy in this population.

## Background

Cancer is a growing health problem [1]. Up to 40% of patients diagnosed with cancer experience clinically significant levels of psychosocial-spiritual distress, often related to the profound life changes associated with a cancer diagnosis, the symptoms associated with the disease itself, and treatment side effects [2, 3]. Such distress can result in serious and far-reaching negative sequelae: poor quality of life, symptoms of depression and anxiety, and poor psychological well-being [4, 5]. The psychosocial-spiritual distress associated with cancer can be addressed using psychosocial and spiritual interventions such as dignity therapy, cognitive-behavioral therapy (CBT), and meaning-centered psychotherapy [6]. These psychosocial and spiritual techniques can improve quality of life, symptoms of depression and anxiety, and spiritual well-being.

Cancer care requires attention to psychosocial-spiritual concerns to support patients’ successful adjustment [7]. However, lack of cultural and spiritual sensitivity in psycho-oncologic care can impact low-uptake among culturally and spiritually diverse groups [8]. Culturally and spiritually sensitive psycho-oncologic care embraces patients’ self-reported stories, beliefs, values, and practices shaped by historical and geopolitical contexts and religious and spiritual beliefs and practices [9]. However, few cancer-care specific psycho-oncologic interventions are specific to Muslim patients [10].

Islam is the second largest and fastest growing religion in the world. By 2050, Muslims will comprise almost 30% of the world’s population; it will be the world’s largest religious population [11]. In 2019, the global Muslim population was estimated at 1.9 billion. The ongoing growth of the Muslim population, and the increasing cancer prevalence among Muslims worldwide, warrant the need to gain insights into psycho-oncologic care, particularly psychosocial and spiritual approaches, used in the treatment of Muslims. The outcomes of psycho-oncologic approaches in patients with cancer have been widely studied and reviewed. Yet, these reviews have not examined such approaches as provided to Muslims. Given the predominance and ongoing growth of Islam, it is necessary to understand Muslims’ experiences with, and perceptions of, psycho-oncologic approaches, particularly with regard to their psychosocial-spiritual care. Thus, in addition to describing the effect of psychosocial and spiritual interventions on mental health, spiritual, and quality of life outcomes, a review is needed that integrates the literature on Muslim cancer patients’ experiences of these interventions.

The purpose of this review was to integrate the literature on the mental health, spiritual well-being, and quality of life outcomes with the perspectives of Muslims who have been treated with psychosocial and spiritual techniques to treat the psychosocial-spiritual distress associated with cancer or its treatment. We used the biopsychosocial-spiritual model as a framework for this review [12]. According to this model, illness can disrupt biological relationships that in turn disrupt the patients’ psychological, social, and spiritual relational aspects. Following the model, culturally and spiritually sensitive cancer care must address the totality of the patient’s relational existence—physical, psychological, social, and spiritual. In the model, spirituality is construed to have four domains: religiosity, religious coping and support, spiritual well-being, and spiritual need [12].

## Methods

### Design

This integrative literature review used Whittemore and Knafl’s [13] methodology and the PRISMA criteria of quality for reporting reviews [14]. We used the narrative synthesis approach as it allows for the inclusion of studies with different research designs, including qualitative studies, thus providing a better understanding of the potential positive outcomes of using psycho-oncologic interventions from multiple lenses [13]. This synthesis provides extensive literature coverage and has the flexibility to deal with emerging knowledge and concepts.

### Literature search

Table 1 presents the search terms and Boolean operators that were used to build the search strategy. This strategy was developed in consultation with a medical librarian. The search was conducted from September to November 2019 and updated in July 2020.

**Table 1:** Search Strategy

Search	Search terms
Search string 1 2 3	(((((((((Islam[Text Word] OR Muslim[Text Word] OR Islamic[Text Word] OR Arab*[Text Word])) NOT Islam[Author]) NOT Arab[Author]) AND (Cancer OR neoplasms OR oncology OR tumor OR malignancy OR carcinoma OR Sarcoma OR Lymphoma OR leukemia OR tumor OR Blastoma)) AND (Psycho* OR counsel* OR therapy OR Support OR non-pharmacologic* OR nonpharmacologic* OR non drug OR non-drug)) NOT drug) NOT medication) NOT pharmacolog*)Islam or Muslim or Islamic or Arab* Cancer OR neoplasms OR oncology OR tumor OR malignancy OR carcinoma OR Sarcoma OR Lymphoma OR leukemia OR tumor OR Blastoma Psycho* OR counsel* OR therapy OR Support OR non-pharmacologic* OR nonpharmacologic* OR non drug OR non-drug NOT drug; NOT medication; NOT pharmacolog*

*Note*: The search strategies were amended according to each database

#### Inclusion and exclusion criteria

Thus, studies were included if they used psychosocial-spiritual interventions, including those that appealed to religiosity and religious coping, as the primary intervention in cancer care; included Muslim participants with a diagnosis of any cancer; and were published in peer-reviewed journals between 2010 and 2020, in English. This timeframe reflects the last decade of international growth of psychosocial-spiritual interventions for Muslims living with cancer, all of which are the result of a systematic search process. We defined psychosocial-spiritual interventions as addressing mental health problems through systematic, time-limited activities, including those that involved complementary therapies, including those that appealed to religiosity and religious coping; that involved contacts between a cancer patient and a trained healthcare provider who sought to ameliorate cancer-related distress by producing changes in individuals’ feelings, thoughts, attitudes, and behavior; and that included a psychotherapeutic component, such as psychoeducation, therapeutic alliance, counseling, and structured, manualized interventions [15]. Studies were excluded if they were case reports or used observational methods, did not describe the applied psychosocial and spiritual technique/approach/intervention in the methods, and/or included psychopharmacologic agents as part of the intervention.

### Procedures

Two authors (AA and ML) independently screened titles and abstracts using the Covidence systematic review software (https://www.covidence.org), to identify studies for full-text screening. These two authors then independently screened full-text studies to identify studies that fit inclusion criteria. At all stages, disagreements were resolved. The reference lists of the included studies were scanned for further studies.

### Data evaluation

The included studies’ quality was assessed using two criteria, methodological or theoretical rigor and data relevance, on a 2-point scale (high = 1, low = 2). Methodological rigor was assessed according to whether the study’s methodology was explained in such detail that it could be replicated. Data relevance was assessed according to whether the data presented addressed the study’s stated aims. No studies were excluded on the basis of quality; however, more weight was given in analysis to studies with rigor and data relevance rates of 1.

### Data extraction, analysis, and synthesis

Narrative synthesis accounted for differences in intervention approaches, study design, and methodological quality among the reviewed studies. Studies reporting similar outcomes were clustered and discussed together to draw meaningful interpretations of the data.

Interpretations regarding clinical relevance were made regardless of whether studies were statistically powered; however, we only described the intervention as useful for studies that used a 2-arm design. We only described the intervention as useful if the outcome measure between the study arms was reported as significantly different (*p* ≤ 0.05).

Extracted data were compared word-by-word in a data extraction table in Excel. We (AA and ML) created a list of keywords for each study. These keywords were reviewed to decide what concepts the data reflected. We used these concepts as codes. Each code was compared to all other codes. Comparisons for similarities, differences, and general patterns were made. Similar and reciprocal codes were categorized and grouped. These coded categories were compared and contrasted. The initial subgroup classification relied on psychosocial-spiritual outcomes, which were analyzed by evaluating all interventions and qualitative designs. We (AA and ML) organized these subgroups by themes based on commonality, relationships, and patterns and refined these themes to encompass as much of the data as possible. Presentations of primary source data were employed to simplify the distinctions between patterns, themes, and associations. We assembled the analogous variables next to one another to assess for any associations between them. The final stage involved a shift from interpretive efforts to descriptive ones that sought to determine patterns and relationships to understand higher abstraction levels.

### Registration

The review methodology was submitted to PROSPERO (International Prospective Register of Systematic Reviews) in August 2019, and was approved (PROSPERO 2020 CRD42020159191; https://www.crd.york.ac.uk/prospero/display_record.php? ID=CRD42020159191).

## Results

### Search outcomes

**Fig. 1:**
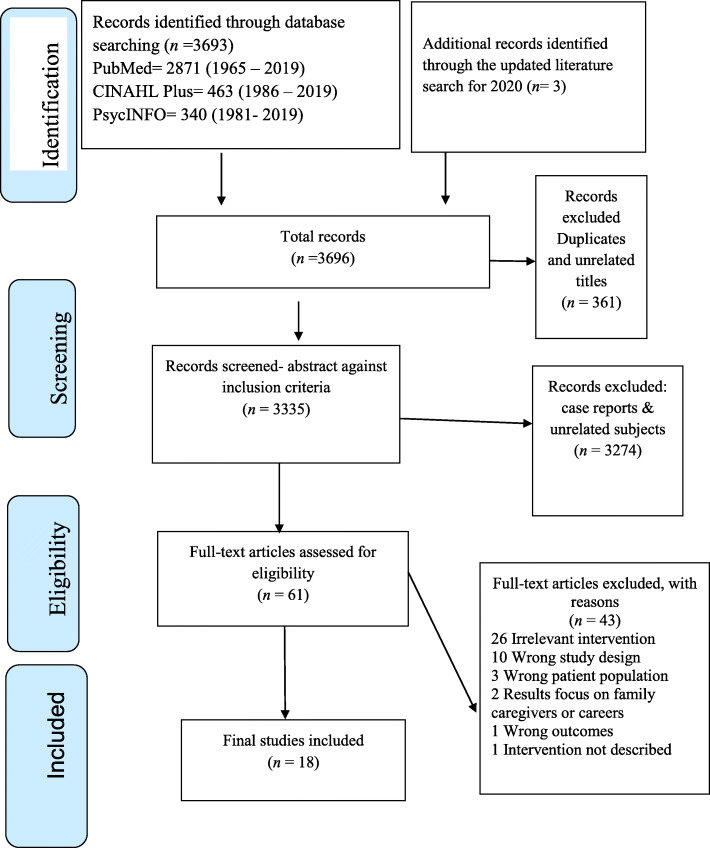
PRISMA flow diagram

The final sample included 18 articles for review (see Fig. 1).

### Study characteristics and quality of the reviewed studies

The included studies in this review were published between 2013 and 2019. The 18 studies included a total of 2996 participants. Female participants outnumber male participants, and all participants’ ages were ≥ 18 years old. Fewer than 50% of the studies used two or more self-reported outcome measures. Table 2 describes the country where the study was conducted and the study’s design and analytic approach.

**Table 2:** Country of Origin and Research Designs of Included Studies

	Number of Studies	Percentage (%)
Country of origin		
Country		
Iran	13	72.2%
Malaysia	2	11.1%
Turkey	2	11.1%
Pakistan	1	5.6%
Total	18	100%
Research designs		
Quantitative studies		
RCT	8	50%
Quasi-experimental	6	37.5%
Control pretest-posttest	1	6.25%
Pretest-posttest without control groups	1	6.25%
Total of quantitative studies	16	100
Qualitative studies		
Thematic analysis-case study	1	50%
Content analysis	1	50%
Total of qualitative studies	2	100%

Table 3 shows the characteristics and findings of psychosocial-spiritual interventions by treatment approach*.* The cancer types and stages in the included studies were heterogeneous and ranged between the early and late cancer stages. However, several studies did not specify the site or stages of cancer of the participants. The most prevalent cancer type in the review sample is breast cancer. These studies were conducted in different cancer care settings, such as hospitals, cancer research centers, and oncology and radiotherapy departments.

**Table 3:** Characteristics and Findings of Psychotherapeutic Interventions by Treatment Approach

Authors & year	Purpose	Design	Country/ Settings	Population		Description of intervention or treatment	Key Findings/ outcome measures	Comments	Quality 1 = high; 2 = low	Intervention category
				Sample characteristics	Cancer type / stage					
17	To evaluate the effectiveness of laughter yoga on the mental well-being of cancer patients undergoing chemotherapy.	Randomized, double-blind, clinical trial	Iran/ Medical Center	69 patients with cancer 67.7% females 47.1% digestive cancer Mage = 49 years	Digestive Breast Respiratory Reproduction Bone/ Not specified	Four 20–30 min laughter yoga sessions consisted of 15 steps: “clapping in rhythm with chanting of ho-ho-ho,ha-ha-ha, deep breathing, warming up and stretching the neck and shoulders, hearty laugh technique, greeting laughter technique, appreciation laughter technique, one-meter laughter technique, silent laughter technique, humming laughter technique, swinging laughter technique, lion laughter technique, cell phone laughter technique, argument laughter technique, gradient laughter technique, heart to heart laughter technique, laughter yoga exercises.”	Warwick-Edinburgh Mental Well-being Scale (WEMWBS) There were statistical differences between the intervention group and the control group in the three dimensions of mental health scores (optimism, positive relationship, and vitality).	A low number of the chemotherapy sessions thus low number of laughter yoga sessions	1	Complementary
18	To investigate the effectiveness of lavender oil aromatherapy on anxiety and sleep quality in patients undergoing chemotherapy.	Randomized controlled trial	Turkey/ Private hospital	70 patients with cancer, Mage = 58.22 years. a large percentage of participants women, married, and with graduates of primary school	Breast, lung, urothelial, ovarian, gastrointestinal, and renal/ Not specified	Lavender oil and tea tree oil (aromatherapy) were administered to the participants in the experimental group. Three drops of aromatherapy were placed on a piece of cotton near the nose.	State-Trait Anxiety Inventory Pittsburgh Quality Sleep Index (PSQI) The authors found a statistically significant improvement in the sleep quality and anxiety state in the lavender group.	Inability to blind the nurse and patients to the aromatic oils used Not stated what participants received in the control group Not clear if all participants are Muslims	2	Complementary
19	To evaluate the effect of chemotherapy counseling on self-esteem, anxiety, and depression of cancer patients.	Randomized control trial	Malaysia/ government hospitals	2120 participants with cancer, Age range = 45–65, 58.2% females, 67.1% married, 52.2% with diploma education	Not specified/ Stage 1–4	A “Managing Patients on Chemotherapy” module through an interactive format was administered by a trained pharmacist-in-charge from 3 to 6 weeks. The model’s content includes an introduction to chemotherapy and its related emotions (such as depression, anxiety, and fear).	Patient health questionnaire-9 (PHQ-9), Generalized anxiety disorder-7(GAD-7) questionnaire, & Rosenberg self-esteem scale (RSES) A significant difference in depression, anxiety, and optimism scores for both groups over time (1st, 2nd, and 3rd follow-up counseling sessions) in the intervention group were reported.	Low attrition rate Large sample size No controlling to participants in the control group with exposing to different education sources	1	Psychoeducation
20	To evaluate the impact of individualized patient education and emotional support on the quality of life of breast cancer patients undergoing chemotherapy.	Quasi-experimental design	Pakistan/ Public hospital	50 patients with breast cancer, 78% 40 years and above, 66% married	Breast Cancer/ Stage 2, 3	A clinical oncologist nurse specialist conducted an intervention for 6 weeks that included four stages: in-person oral and written patient education (in a booklet), the nurse visiting the patients during chemotherapy administration (the nurse re-evaluate patients’ reported concerns, analyzed their improvement, and implemented accordingly), follow-up phone calls with the patients or their family members (the nurse evaluated patients’ problems and intervened accordingly), and the patients and family members’ reached out to the nurse via phone (the nurse addressed the patients’ problems or referred them for seeking medical help).	The Functional Assessment of Cancer Therapy-Breast (FACT-B version 4) The intervention group participants scored improvement in the overall quality of life and emotional well-being compared to the control group.	Small sample size Lack randomization Conducting the intervention and collecting data by the same nurse leading to research bias Temporal bias because of non-randomized time blocks between recruitment of the experimental and control groups Not clear what the control group was Women participants only	1	Psychoeducation
21	To assess a chemotherapy counseling module’s effect on quality of life and psychological outcomes among cancer patients.	A single-blind randomized controlled trial	Malaysia/G government hospital	162 patients with cancer, Mage = 67.46, 58% females, 66.7 married, 47% with diploma education	Not specified, stage 1–4	The therapy module (45 min ±10 min) focused on different topics, including education about the psychological aspects of patients undergoing chemotherapy, such as depression and anxiety	WHO quality of life-BREF (WHOQOL-BREF), Patient health questionnaire-9 (PHQ-9), & Generalized anxiety disorder-7 (GAD-7) Statistical differences in the overall quality of life (physical health, mental health, social relationships, and environment) and psychological effects (anxiety and depression) between the study populations were reported.	Using self-reported measures participants recruited from one hospital which limits generalization	1	Psychoeducation
22	To examine the effectiveness of CBT and spiritual-religious intervention in enhancing coping responses and quality of life among breast cancer survivors.	Quasi-experimental trial of pre-post-test study	Iran/ Cancer Research Center	45 survivors, Mage = 45.15, 80% married, & 60% with diploma education	Breast Cancer/ Clinical stage 1, 2, 3	Cognitive-Behavioral Therapy (CBT) & Spiritual-Religious Intervention (SRI)- Eight sessions The authors followed the CBT protocol developed by Kvillemo and Branstrom [16]. The first sessions included a preface about the CBT, assignments, “diaphragmatic breathing practices,” relaxation techniques. The middle sessions encompassed assertiveness, stress management, problem-solving, and identification of negative thoughts. The final session focused on reforming negative thinking and review the contents. A Cleric man, an expert in psychology, revised a model proposed by Richards and Bergin [17] to guide the spiritual-religious intervention for this study. This intervention included eight sessions, which begin with an introduction to the intervention and practicing meditation. Sessions 2 to 5 focused on practicing Zekr (holy words repetition), Doa (prayer), Tawakkol (trust in God), and Sabr (patience). During these sessions, a discussion about the association between these religious practices and mental peace was conducted. In the last sessions, self and others’ forgiveness was argued, and a revision for the intervention sessions was conducted.	Coping Response Inventory & QLQ-C30 Although no statistically significant findings were reported in the control and experimental groups, both interventions were found to be effective in improving the quality of life and coping responses of survivors.	No controlling for some confounding variables “such as the physical, mental, economic, and socio-cultural status of participants” Limitation in generalizing the study results Small sample size Waitlist control group The control group participants did not receive any psychological treatment until the experimental group ended treatment and collected post-intervention data.	1	CBT
26	To test the effect of mindfulness-based cognitive therapy on posttraumatic growth, self-management, and functional disability among patients with breast cancer.	Randomized controlled trial with repeated measures design	Iran / Department of Oncology and Radiotherapy	20 women with breast cancer, Mage = 38.8 years, 60% with diploma education, 80% homemaker	Breast cancer / Stage 2	Eight sessions of mindfulness-based cognitive of 2.5 h each Williams and colleagues’ (2016) manual of Mindfulness-Based Cognitive Therapy was used to conduct the study intervention. This intervention included different strategies, including assignments, confronting obstacles, re-examining previous session activities. The first sessions encompassed “an automatic guidance system,” creating relationships, providing feedback, and discussing body exercises and mindfulness meditation. The middle sessions included breathing mindfulness meditation, distributing materials about meditation, practicing breathing, sitting meditation, mindfulness of breathing, and determining patients’ responses to stress and its link to pain. These middle sessions also included learning differences between thought and fact, conscious yoga, relaxing, and meditation sessions. The last sessions involved testing and discussing the program, clarifying the significance of self-care, repeating previous activities, naming enjoyable activities, and distributing brochures.	Structured Clinical Interview for DSM-5, Posttraumatic Growth Inventory (PTGI), Patient Activation Measure and WHO Disability Assessment Schedule 2.0 The findings demonstrated that the effect of mindfulness-based cognitive therapy on psychosocial aspects (self-management, posttraumatic growth, and functional disability) was statistically significant in the post-test (*P* < 0.008) and follow-up (*P* = 0.014) between the intervention and control group.	Small sample size Participation limited to married women, which limits the generalization of the study findings	1	CBT
25	To examine the effectiveness of mindfulness-based cognitive therapy on relief of hopelessness symptoms among women with breast and gynecological cancer.	Pretest-posttest with group-control group design	Iran/ Cancer Research Center	82 patients, age range = 24–65 years	Breast and gynecological cancer/ Not specified	Eight weekly mindfulness-based cognitive therapy sessions were conducted with a focus on guided relaxation and mindfulness meditations, group discussions, psychoeducation (about cancer, mindfulness, relaxation techniques and exercises, and cognitive restructuring skill development), and homework assignments.	Beck Hopelessness Scale (BHS) Mindfulness-based cognitive therapy had a statistically significant effect on hopelessness, and there was a significant difference between the study arms in terms of hopelessness due to loss of motivation.	Lack randomization	1	CBT
24	To compare the effect of the metacognition treatment (MCT) and mindfulness-based cognitive therapy (MBCT) on anxiety, depression, and stress in females with breast cancer.	Quasi-experimental pretest and posttest study with control group	Iran/ General hospitals for breast cancer	36 patients with cancer, age range = 38–49 years	Breast cancer/ Not specified	Eight sessions of mindfulness-based cognitive therapy were conducted with an introduction about “automatic guidance system/knowledge on how to use present moment awareness of bodily sensation”, thoughts to alleviate stress, homework, distributing educational material about meditation and yoga practices, examining of body exercises, practicing breathing in mindfulness meditation. The middle sessions focused on re-practicing sitting meditation, identifying participants’ reactions to stress, and practicing knowing about sounds and thoughts. Participants in the last few sessions practiced mountain meditation, listed some exciting activities, checked physical exercise, and discussed procedures. The metacognition treatment included introducing this intervention, practicing strategies for enhancing attention, homework, identifying negative thoughts, practicing detached mindfulness, and examining uncontrollable beliefs. The middle sessions focused on re-examining uncontrollable beliefs, challenging with positive beliefs about rumination, identifying negative thoughts. The last sessions addressed negative beliefs and useless strategies, remodeling recurrent fears, examining the other cognitive beliefs, and discussing using a new program.	Depression, Anxiety, and Stress Scale - 21 items (DASS-21) questionnaire The authors reported a statistically significant difference in the rate of depression, anxiety, and stress in the experimental, post-test, and follow-up group.	Small sample size Limitations in generalization of the findings Women only	1	CBT
27	To evaluate the effect of the mindfulness-based stress reduction program and conscious yoga on women’s mental fatigue severity and life quality with breast cancer.	Quasi-experimental study with a pre-test, post-test, and control group	Iran/ Division of Oncology	24 patients with breast cancer, 30 to 55 years/ Mage = 44.8 ± 3.28	Breast cancer/ stages 1,2, 3	The Mindfulness-Based Stress and conscious yoga program consisted of 8-week in which every session lasted about 2 h. The first third sessions of the Mindfulness-Based Stress Reduction Program included an introduction for “automatic guidance system/knowing how to use present moment awareness of bodily sensation,” alleviating thoughts associated with emotions, feedback, homework, deep breathing exercise, discussion about examining body exercise, practicing breathing mindfulness meditation/ yoga stretching exercise, sitting meditation), and videotape of yoga practices. The middle sessions focused on practicing conscious yoga, awareness of breathing, body, sounds and thoughts, sitting meditation. The final sessions included practicing mountain meditation/sleep hygiene/ repeating exercises of the previous session/making a list of enjoyable activities. In all sessions, educational materials were distributed for each session.	Fatigue Severity Scale, Global Life Quality of Cancer Patient, and Specific Life Quality of Cancer Patient questionnaires Results showed that mindfulness-based stress reduction treatment significantly enhanced the overall quality of life (cognitive, emotional, and social functions) in the interventional group compared to the control group. At the end of the study, participants in the control group were provided with CDs of yoga exercises.	Small sample size Lack of contextual and individual factors control Women only	1	CBT
23	To evaluate occupational therapy’s effectiveness in fostering the quality of life for men who treat Metastatic prostate cancer.	Randomized controlled study	Turkey/ Department of Occupational Therapy	34 patients, age ≥ 50 years	Prostate cancer/ Stage 3 & 4	Participants in the intervention group (cognitive behavioral therapy based occupational therapy) received daily training about self-care, productivity, and/or leisure. The intervention lasted 12 weeks and included 30-min recreational activity; 40-min didactic cognitive behavioral therapy based on occupational therapy education and information on the diagnosis and treatment of metastatic prostate cancer; 20 min of practical relaxation skills training.	The study indicated that participants’ quality of life increased significantly in statistical terms for the interventional group, compared to the participants’ results in the control group. Interventionists prompted participants in the control group to practice independent self-care, recreational group activities, relaxation techniques in daily life outside of hormonal therapy. Participants were provided with a printed home program after the initial assessment and instructed on these activities’ effects.	The lack of control at home Small sample size Limitations of the generalizability of the findings to those who have no significant cognitive impairment	1	CBT
10	To evaluate spiritual psychotherapy’s effectiveness, emphasizing prayers’ significance on psychological health and pain in cancer patients.	Quasi-experimental study	Iran/ Not specified	76 patients with cancer, 64.9 females, 51.4% married, & 32.4% with middle school education	Not specified	The religious psychotherapy focused on presenting the impacts of religious attitudes, and the investigator assisted the patients in promoting participants’ religious beliefs. These meetings also focused on wisdom, God’s mercy, and hope for God’s mercy. The participants prayed and were directed to consider prayer’s meaning to adopting proper religious strategies to improve psychological health using the *Quranic* instructions and the mental mechanisms existing in these prayers. Richards and Bergin’s religious principles were utilized to focus on Islamic perceptions, including prayer, reciting Holy books, visiting religious places, and forgiving others.	GHQ-28 General Health Questionnaire The authors found that the mean and standard deviation of mental health scores after the intervention were statistically significantly increased.	Small sample size Lack randomization	1	Religious principles and practice
16	To explore the structure of spiritual counseling sessions among patients who experience chemotherapy.	Qualitative descriptive thematic analysis-case study	Iran/ oncology departments	22 patients, age range = 18 to 85, Mage = 53 years, 16 females and 6 males	Breast, gastrointestinal, & blood cancer/ Not specified	Through spiritual counseling, the counselor implemented strategies to settle the patient’s problems, depending on faith and spiritual powers. Counselors urge patients to talk about their religious or spiritual problems and concerns.	Four themes emerged: (1) “history-Taking”: The counselor asked questions on religious and spiritual behaviors first and then about cognitive behaviors. This aspect addresses the relationship between the patient and God, the patient’s beliefs, and religious practices and teachings during the illness and hospitalization period. (2) “general advice”: The spiritual counselor afforded general advice to solve the patient’s challenges and concerns in various fields. The advice involved hope for healing, promotion of self-confidence, and the necessity for self-care, which occasionally consist of mental health care. (3) “spiritual-religious advice”: Spiritual-religious advice encompassed reciting many verses and stories linked to the disease. The counselor addressed the causes of illness and adversity from the perspective of religion, spiritual reinforcement after the diseases, and faith in everlasting life. The counselor recommended them to trust in God and to pray for the Creator. Other topics were related to some religious teachings and practices and resolving religious issues associated with the means of worship. (4) “dealing with patients’ spiritual or religious ambiguities and paradoxes”: Patients often face contradictory situations, especially in the case of existing conflict between the patients’ beliefs and counselor’s interpretations. Some situations could be vague for the patient, such as spirituality and the philosophy of praying. Other ambiguous situations include the patient’s challenges regarding God and the validity of the said stories.	Small sample size Limitations in generalizing the findings Single arm case analysis	2	Religious principles and practice
31	To evaluate spiritual group therapy’s effect on quality of life and spiritual well-being among patients with breast cancer.	Quasi-experimental pretest-posttest study with a control group	Iran/ General hospital	24 participants with breast cancer, 57% under 50 years old, 79% married, 50% with high school diploma	Breast cancer/ Not specified	The intervention included 12 sessions, with a focus on spiritual group therapy. The initial meeting included the following activities: meeting members, discussing spiritual and religious beliefs and their impact on life, listening to the inner voice, and communicating with God or any superpower. The middle sessions addressed altruism, holy relationships, resentment, and lack of forgiveness, feeling guilty, and forgiveness. The final sessions focused on death and fear of death, faith and trust in God, and attitude and thanksgiving. The module focused on different topics, including education about patients’ psychological aspects undergoing chemotherapy, such as depression and anxiety.	Structured Clinical Interview for DSM-IV (SCID-I), Quality-of-Life Questionnaire (WHOQOL-26), and Spiritual Health Scale (SWB-20) There are statistical differences in the quality of life and spiritual well-being between the study groups.	Small sample size Lack randomization Inadequate experience in treating patients with cancer using group therapy and spiritual therapy The control group did not receive any spiritual therapy until the experimental group was fully treated	1	Religious principles and practice
30		Randomized clinical trial	Iran/ referral center for cancer	42 women with cancer, Age range = 26–74 years, 78.6% married, 38.1% illiterate, 45.2% with breast cancer	Breast cancer, gastrointestinal, and other types/ Early stages	Trained counselors, under the supervision of clinical psychologist, conducted individual intervention of spiritual counseling eight times a week. These sessions included educational materials about Islam as a part of the intervention. The spiritual counseling encompassed a question and answer period, sharing, reflecting, providing feedback, homework about reciting of Holy *Qur’an*, relaxation exercises, and meditation.	Spiritual Well-Being Scale The authors found statistically significant differences in spiritual well-being scores between the study arms post-intervention.	Small sample size No follow-up after the intervention, limitation in generalization the findings beyond Shiite Muslim Women with cancer	1	Religious principles and practice
28	To examine the effectiveness of spiritual/religious intervention on the illness perception of women with breast cancer.	Pretest-posttest design	Iran/ not specified	45 patients, 37 to 58 years/ Mage = 43.68, 34.1% with high school, 95.1% married	Breast cancer/ Not specified	Ten sessions were conducted that focused on Islamic religious practices and beliefs, building communication bridges, analyzing the patients’ views, expressing the benefits and effects of spirituality on health, asserting religion’s belief in the purposefulness and value of life, and the benefits of hope. The sessions also included positive thinking, optimism, and vitality, expressing the causes and outcomes of disasters, and strengthening faith.	Illness Perception Questionnaire Spiritual intervention has a positive impact on the enhancement of illness perception in women with breast cancer.	Small sample size Limitation in generalization The intervention had too many elements and not clear what the moderator was (positive thinking, optimism, or religious practices and beliefs) The control group received the usual medical care Only women	1	Religious principles and practice
29	To explore patients’ experiences about spiritual care and spiritual interventions among patients living with cancer.	Qualitative content analysis	Iran/ Oncology units	10 patients with cancer, age range = 20–61 years, 4 women and 6 men	Leukemia, breast cancer, colon cancer, lymphoma, sarcoma, stomach cancer, and liver cancer/ Not specified	–	Patients regarded their beliefs in God is the main source of their power, and this source promotes their inner strength contesting the fears of death through the close relationship and trust in God. Patients believe in Islamic beliefs about life, death. The absolute faith in God and strengthen this faith among the participants resulted in more coping responses to cope with cancer and its associated problems. Patients pray for the aim of healing and seek that from God and through people who have superior status among Muslims like Holy Imams. Most Muslim patients in this study used a strategy of acceptance of divine providence in which they bolster spirituality dimension faith in the divine providence. They believe that each person has a destiny from God and is in God’s hands, which precludes them from getting frustrated.	Limitations to generalize findings to other areas and cultures Small sample size	2	Religious principles and practice
15	To evaluate the effectiveness of spiritual therapy intervention in improving Iranian women’s spiritual well-being and quality of life with breast cancer.	Randomized controlled clinical trial	Iran/ Cancer Research Center	65 women with breast cancer, 47.9 years, 95.3% married, & 50.7% homemakers	Breast cancer/ Not specified	Over six sessions, each lasting 20–30 min, the spiritual therapy intervention included a theme embracing guided relaxation, and meditation exercise, exploring the negative and positive thoughts, and prayer therapy. Participants received a manual and a CD-ROM containing materials and PowerPoint slides covered in these sessions.	Functional Assessment of Chronic Illness Therapy Spiritual Well-being scale (FACIT-Sp12) and cancer quality-of-life questionnaire (QLQ-C30) After six sessions of the intervention, a statistical significance was reported regarding improving spiritual well-being and quality of life between the study’s arms (푃 < 0.001). Patients in the control group received standard management and treatment and routine educational program.	Small sample size Lack of an attention control group No follow-up after the intervention Women only	1	Religious principles and practice

The psychosocial-spiritual interventions’ duration ranged from 3 to 12 weeks. The contents of the interventions and duration and length of sessions varied, but two had relatively similar content and protocol [18, 19]. A trained facilitator performed the interventions in some of the included studies (*n* = 5, 28%). Two studies [20, 21] used complementary therapy such as laughter yoga and aromatherapy. Three studies implemented psychoeducation, including education about the emotional and psychological aspects [22–24]. Six studies [25–30] used different strategies for cognitive behavioral therapy (CBT). Finally, seven studies [10, 18, 19, 31–34] relied on Islamic religious principles and practices as a part of a psychosocial-spiritual intervention among cancer patients that had a psychotherapeutic component to it. The included studies evaluated mental health outcomes using different scales, such as the Warwick-Edinburgh Mental Well-being Scale (WEMWBS), Generalized Anxiety Disorder-7(GAD-7), and the Rosenberg Self-Esteem Scale (RSES). The quality scores and comments on reviewed studies are presented in Table 3.

### Themes

Four major themes emerged: (1) Treating Mental Health Without Psychopharmacologic Agents, (2) Improving Knowledge of Cancer for Improving QOL, (3) Depending on Faith for Spiritual Well-being, and (4) Relying on Religious and Spiritual Sources: Letting Go, Letting God.

### Treating mental health without psychopharmacologic agents

Among the 18 studies, eight studies showed improvement in mental health outcomes [10, 20–22, 24, 27, 29, 34]. Specifically, psychoeducation [22] and CBT [27] improved depression and anxiety symptoms and stress. Mindfulness-based cognitive therapy had a significant long-term effect (*p* = 0.014) on self-management, posttraumatic growth, and functional disability [29]. Aromatherapy [21] contributed to stress relief and improved sleep quality. Complementary therapy (laughter yoga) [20] and psychoeducation [22, 24] enhanced a sense of optimism and hopefulness.

### Improving knowledge of Cancer for improving QOL

This theme was represented by seven studies that addressed the role of CBT [25, 26, 28, 30], spiritual therapy [34], and psychoeducation [23, 24] on enhancing quality of life in Muslim patients with cancer. Spiritual group therapy helped patients listen to their inner voice, let go of resentment, and forgive, which led to improvements in quality of life [18, 34]. Education about the psychological aspects of cancer and mindfulness-based stress reduction [30] assisted patients in enhancing cognitive, emotional, and social function [24]. Other participants showed improvement in overall quality of life and emotional well-being among psychoeducation groups [23]. Men with prostate cancer also indicated that CBT enhances their quality of life [26]. Finally, a combination of CBT and a spiritual-religious intervention was found to promote breast cancer survivors’ quality of life and coping responses [25].

### Depending on faith for spiritual well-being

Five studies employed a spiritual therapeutic technique [18, 19, 32–34], all of which reported improved spiritual well-being. Patients who received spiritual psychoeducation and counseling, such as educational materials about Islam, relaxation exercises, and meditation, reported improved spiritual well-being scores [33]. Women with breast cancer, through spiritual therapy, discussed spiritual and religious beliefs (regarding death and fear of death, faith, and trust in God) and the effect of these beliefs on life [34]. In Jafari and colleagues’ [18] study, women with breast cancer explored negative and positive thoughts in a spiritually based therapy, which resulted in improved senses of meaning and peace.

### Relying on religious and spiritual sources: letting go, letting god

Muslim patients with cancer relied on spiritual and religious sources while applying psychosocial-spiritual therapy to provide comfort, coping, and meaning in their experience, as described in three studies (spiritual counseling [19] & spiritual-religious interventions [23. 30]). Patients living with cancer considered their belief in God as a central source of their power [32]. This source supported their inner-strength, which was necessary to fight death anxiety. Patients adopted a strategy of accepting divine providence, which leads to improvements in the faith element of spiritual well-being. In the 2017 study by Ghahari and colleagues [25], breast cancer survivors used spiritual/religious resources to solve personal and interpersonal problems that enhanced their coping responses. Both were practicing prayer and religious advice, such as reciting verses from the *Qur’an*, which played a paramount role in alleviating patients’ suffering and promoting a sense of contentment and self-confidence [20].

## Discussion

The purpose of this integrative review was to synthesize the research on the psychosocial-spiritual outcomes of psychosocial-spiritual interventions in Muslim patients undergoing treatment for cancer. We used a narrative approach to research synthesis and sought to generate new insights and recommendations by going beyond the summary of findings from different studies [14]. The individual studies used various outcome measures, cancer types and stages, and intervention modalities in our review. This heterogeneity renders it challenging to conduct a systematic review due to clinical diversity (population, intervention, & outcomes), inconsistency in effect size and direction, and lack of data to calculate standardized effect sizes, hence the narrative design.

Psychosocial-spiritual interventions are nonpharmacological strategies that address psychosocial-spiritual distress associated with cancer [5]. Throughout this review, we noted a myriad of psychosocial-spiritual interventions studied in Muslim patients with cancer that target various psychosocial-spiritual outcomes, including promoting patients’ mental health, quality of life, and spiritual well-being [24, 25], which were the most common outcomes in the studies included for review. Reviewed studies have shown that CBT-based interventions are promising strategies to improve psychosocial-spiritual outcomes in Muslim patients with cancer [10, 18, 19, 31–34]. The reviewed studies are also informative in building a base for the effectiveness of psychosocial-spiritual interventions in Muslim patients’ psycho-oncologic treatment.

This review confirms the positive outcomes of various psychosocial-spiritual interventions on improving mental health, such as improving symptoms of depression, anxiety, and stress [10, 20, 21, 24, 27, 29, 32, 34]. Consulting sessions [23, 25] provide patients with practical and educational information and resources related to emotions such as depression, anxiety, and fear associated with cancer. Mindfulness-based cognitive therapy [27, 29] increases patients’ awareness of their feelings; throughout this therapy, patients acquire cognitive skills that promote metacognitive awareness, acceptance of negative thoughts, and an ability to effectively cope with psychological distress. Aromatherapy [21] entails using volatile essential oils of plants to enhance mental health. These oils stimulate the olfactory nerves, which connect to long-term memories that involve long-forgotten memories and their emotional links to one’s life. These emotions can enhance sleep quality and relieve stress. Laughter yoga [20] includes various techniques, such as clapping and chanting, and deep breathing, which prepare the mind for happiness and improve a sense of optimism and hopefulness.

This review also suggests that different psychosocial-spiritual interventions can enhance Muslim cancer patients’ quality of life. A diagnosis of cancer and its associated treatment leads to emotional distress because of deteriorating health and impending death, which can result in reduced quality of life. The hopelessness [18] that is associated with poor quality of life can also be a predictor of depressive symptoms among patients with cancer. Seven of the studies included in this review suggest that psychosocial-spiritual strategies can improve patients’ quality of life [23–26, 28, 30, 34].

Mindfulness-based cognitive therapy [28] helps patients by incorporating cognitive therapy and meditative practices to attract attention to thoughts and feelings without prejudging consciously. This can help patients to improve mood and combat depressive symptoms such as hopelessness, and in turn, enhance quality of life. While yoga sessions [30] and psychoeducation [23] may stimulate brain pleasure centers, spiritual therapy [31] works on promoting illness perception through patients’ cultural beliefs and psychological needs. Zamaniyan and colleagues [34] indicate how spiritual therapy that includes education about the psychological aspects of patients undergoing chemotherapy contributes to improving symptoms of depression and anxiety, ultimately enhancing patients’ quality of life.

Some authors discussed the role of spiritual counseling and therapy [18, 19, 32–34] in improving spiritual well-being. These approaches help patients to increase self-awareness and broaden inner strengths and resources through addressing their spiritual questions, reciting *Qur’an*, and practicing relaxation exercises and meditation. Rassouli and colleagues [32] used these approaches to support patients coping with cancer and its related problems. Patients’ religious beliefs and some practices may conflict with therapists’ interpretations of patients’ experiences. Therefore, these spiritual counseling approaches may help patients with cancer to find meaning in the cancer experience and resolve these conflicts [19, 33]. Finally, Jafari and colleagues [18] demonstrate how a spiritual therapy intervention can help patients identify and shift negative thoughts and validate positive ones.

In three studies [19, 25, 32], the participants believed that God has the power to control their lives and circumstances and that God alone can cure the disease. These participants attributed their cancer to the will of God and admitted that they could not alter their own fates. These beliefs may help observant Muslims cope with negative feelings and experiences that may be associated with cancer. Patients acknowledged the significance of their absolute belief in God’s forgiveness and mercy as religious practices and spiritual resources and, while applying psycho-spiritual therapy, support the process of changing feelings of powerlessness into feelings of power.

There may be belief-hurdles for some Muslims. For example, they may feel that God has preordained all that happens in life, even cancer. Others may feel that, sometimes, suffering redeems for past sins. These belief-hurdles should be addressed in future studies, including how prevalent they are among Muslims, as they may be more culturally rather than theologically bounded.

### Implications for research

The psychosocial-spiritual approaches in the included studies were not all described with the specificity necessary for replication. Psychosocial-spiritual approaches already established as efficacious in cancer patients need to be adapted to be culturally and spiritually sensitive to Muslims undergoing treatment for cancer and then tested to determine these adaptations’ benefits in this understudied population. And rigorous research designs, such as sufficiently powered randomized control trials with well-structured control groups, are necessary. Measuring the effects of extant efficacious psychosocial-spiritual interventions using a common set of standardized mental health, quality of life, and spiritual well-being outcome measures will facilitate comparing and synthesizing results of different studies across populations.

Our findings stress the need to conduct further randomized control trial (RCT) research with larger sample sizes of participants to determine the benefits and efficacy of culturally and spiritually sensitive psycho-oncologic interventions. RCT research would help to draw more definite conclusions about the efficiency of psycho-oncologic interventions. This will require standardized protocols for culturally and spiritually sensitive psycho-oncologic interventions, such as cancer type and stage included, uniform durations, and topics covered across the population. Cohort studies are also needed to evaluate the level of stability of these therapies’ long-term effects and improve the science of psycho-oncologic interventions. In conducting these studies, researchers should consider the representation of cancer patients from different socio-economic, cultural, and religious backgrounds to develop more sensitive adaptations of psychosocial-spiritual approaches to the care of Muslims living with cancer.

In addition, further qualitative studies are needed to explore the psychosocial-spiritual needs of cancer patients of different ages, cancer stages, and ethnicities. As well, none of the included studies reported cost or examine cost-effectiveness analysis, which is a crucial matter that should be considered in developing countries.

There is a paucity of studies conducted in the Middle East, the sub-continent of Asia, and the Asia-Pacific region, where most Muslims live. This lack may be due to conditions specific to, or a need to invest resources for rigorous RCT research in, these regions. While there are no religious restrictions on psychosocial-spiritual interventions (and indeed, in our experience the more devout a patient is the more inclined the patient is to accept psychosocial-spiritual interventions), clinicians and some patients may opt for a medical approach, due to quick onset of psychotropic medications’ effects, and that such medications are less costly than psychosocial-spiritual interventions. As well, there is pervasive doubt in the effectiveness of psychosocial-spiritual interventions. Testing interventions in rigorous RCTs may help to change this perception. There is also a paucity of studies conducted among Muslim cancer patients who live in Canada, Europe, the United Kingdom, and the United States. As Muslim populations grow in these areas, psychosocial-spiritual intervention studies in Muslims undergoing cancer treatment will be necessary for these regions.

### Implication for practice

Patients and healthcare providers should work together to evaluate the psychosocial-spiritual distress associated with cancer and provide culturally and spiritually sensitive psycho-oncologic care. Since non-Muslim healthcare providers are not fully aware of how to offer culturally and spiritually sensitive cancer care to Muslims, this may result in misunderstandings of their religious beliefs and practices [17, 35]. Thus, culturally and spiritually sensitive psycho-oncologic interventions are likely to improve Muslim patients’ psychosocial-spiritual outcomes. Cultural and spiritual diversity is a variant that needs to be considered when teaching non-Muslim providers [16, 36]. Since psychosocial-spiritual approaches differ in their contents, durations, and goals, manuals of interventions adapted for, and tested in, Muslims would enable non-Muslim providers to deliver culturally and spiritually sensitive psycho-oncologic care.

Seeking medical help or disclosing psychosocial-spiritual distress because of mental illness stigma may be a matter of great concern among patients [35]. This stigma is not based on religious beliefs and practices, but rather on the stigma that a cancer diagnosis carries [37]. This stigma may interfere with seeking psycho-oncologic help to improve their mental health, quality of life, and spiritual well-being [38]. Culturally and spiritually sensitive interventions may help to reduce this stigma.

Overall, Muslims consider Islam a comprehensive way of life, and their faith plays a vital role in coping with adverse life events. Spirituality has a positive role in coping with loss and disease. As a consequence, non-Muslim cancer care providers need to be educated about culturally and spiritually sensitive psycho-oncologic interventions. Such providers need to learn about patients’ cultural backgrounds, religious beliefs and values (e.g., sincerity and selflessness), social norms (e.g., hospitality and generosity), and hierarchies, such as respect for the seniors, and gender differences. Future psycho-oncologic and palliative care practices require greater clarification regarding spiritual care competencies in an increasingly globalized world.

### Limitations and strengths

Our findings should be considered in the context of their methodological shortcomings and potential limitations in generalizability. The scientific rigor of the studies included varies. The majority of reviewed studies recruited relatively small sample sizes, which resulted in being underpowered to detect the effects of these psychosocial and spiritual interventions. The experimental studies included in this review did not indicate whether intervention fidelity was applied in their protocol, and some lacked randomization, blindness techniques, and control groups. Most reviewed studies did not examine long-term effects, but rather focused on effects 3–12 weeks post-intervention. Only one study examined intervention effects at 10 weeks [31], and two studies at 12 weeks [26, 34]. The included studies used various controls, outcome measures, and intervention modalities, which rendered synthesis across studies challenging. This diversity makes it difficult to draw conclusions about any specific modality for a particular cancer stage or type. Our review highlights the importance of future studies sufficiently powered and with long-termfollow-up. We used the procedure of both authors coming consensus regarding disagreements in code development and data analysis [39]; however, code development and data analysis could have been strengthened by using a third researcher to settle disagreements.

Several studies included participants with a range of cancer types and stages simultaneously, instead of focusing on a specific type, stage, or treatment phase. This is a challenge in conducting cancer studies, except at major academic cancer centers where it is possible to conduct studies in patients of only a specific cancer type, stage, or treatment phase. Some studies included in this review did not specify the cancer stage or treatment phase, nor did they specify what the control group participants received. These limitations can act as threats to these studies’ validity or mask the real effect of the interventions implemented. Thus, in addition to well-powered studies with long-termfollow-up, future studies in homogenous populations are needed.

## Conclusion

The reviewed studies provide an overview of the current state of research on psychosocial-spiritual interventions used to address psychosocial-spiritual distress associated with cancer in Muslim cancer patients. It complements previous reviews that did not include Muslims, which are soon to be nearly 30% of the world’s population [12]. Our results indicate the need for increased capacity to address Muslim patients’ psychosocial-spiritual needs living with cancer. Considering the rigor of the studies involved, in addition to their limitations, the evidence discussed here supports future studies to build an evidence base for clinical practice. Incorporating psychosocial-spiritual counseling and therapy into routine cancer care can promote the mental health, quality of life, and spiritual well-being of Muslims undergoing treatment for cancer. Researchers need to further examine the psychosocial-spiritual outcomes of established psycho-oncologic treatment modalities adapted to Muslims. Manualized interventions can help non-Muslim providers deliver culturally and spiritually sensitive cultural psycho-oncologic cancer care to Muslim patients.

## Data Availability

Data are available upon reasonable request to the corresponding author.

## Abbreviations

AA: Abdallah Abu Khait
ML: Mark Lazenby
CBT: Cognitive Behavioral Therapy
QOL: Quality of Life
